# An Investigation of the Relationship Between Pancreas Volume, Nutritional Status, and HbA1c in Geriatric Patients

**DOI:** 10.3390/medicina61040711

**Published:** 2025-04-12

**Authors:** Mercan Tastemur, Cagla Ozdemir, Esin Olcucuoğlu, Muhammed Said Besler, Halil Tekdemir, Gunes Arik, Kamile Silay

**Affiliations:** 1 Department of Geriatrics Medicine, Ankara Bilkent City Hospital, Ministry of Health, Ankara 06800, Turkey; drgunesarik@gmail.com (G.A.); kamilesilay@hotmail.com (K.S.); 2 Evliya Çelebi Training and Research Hospital, Kütahya Health Sciences University, Kütahya 43020, Turkey; cagla_gocen06@yahoo.com.tr; 3 Department of Radiology, Ankara Bilkent City Hospital, Ministry of Health, Ankara 06800, Turkey; esinolcucuoglu@gmail.com (E.O.); msbesler@gmail.com (M.S.B.); haltek04726@gmail.com (H.T.)

**Keywords:** pancreas volume, computed tomography imaging, diabetes, malnutrition, elderly

## Abstract

*Background and Objectives*: With physiological aging, the pancreas is expected to decrease in size as in every organ. The objective of this study was to examine the correlation between pancreas volume (PV), nutritional status, and glycolyzed hemoglobin A1c (HbA1c) in elderly patients with and without type 2 diabetes mellitus (DM). *Materials and Methods*: Between July 2020 and April 2022, 109 patients aged ≥ 65 years who applied to geriatrics clinics and outpatient clinics were included in the study. PV was measured from available abdominal contrast-enhanced computed tomography (CT) scans. Patients were divided into two groups according to the presence of DM. The relationship between PV; biochemical parameters, especially HbA1c; and Mini Nutritional Assessment Short Form (MNAsf) score was analyzed between groups. *p* < 0.05 was considered statistically significant. *Results:* The mean age of all participants was M: 77.40, with SD: 7.32. A total of 54.1% of the participants were female, and 55 had DM. There were no significant differences in age and gender between those with and without DM. Glucose (*p* < 0.001), HbA1C (*p* < 0.001), and triglycerides (*p* < 0.001) were significantly higher, and HDL (*p* < 0.001) was significantly lower in patients with DM. PV was also significantly lower in those with DM (*p* = 0.028). A correlation analysis revealed significant positive correlations between PV, the MNAsf score (rho (109)) = 0.413, *p* = 0.003), and lipase (rho (109)) = 0.297, *p* = 0.002). *Conclusions:* PV, which is expected to decrease with age, was found to be lower in patients with DM in our study in which we evaluated elderly patients with and without DM. We also found that PV was associated with malnutrition. Our study highlights the importance of determining the clinical effects of pancreatic volume in the geriatric population where organ atrophy is expected. Therefore, we believe that more comprehensive studies are needed to clarify the clinical implications of pancreatic volume on our diagnostic and therapeutic decisions.

## 1. Introduction

The global population is experiencing rapid expansion, with the elderly population constituting the predominant demographic segment within this growth. Age is a risk factor for many diseases. As the population ages, health care costs are increasing and becoming a significant burden on society [1]. This is why it is important for individuals to age well. Successful aging is defined as low morbidity and few disease-related sequelae, high physical and cognitive capacity, and active living [2].

Studies in the elderly are important to uncover the unknowns and take necessary precautions. Aging is a process that begins at birth and includes development, maturation, and senescence, with both physiological and pathological aging processes. In physiological aging, there is a decline in organ function in all individuals. Pathological aging is characterized by the formation of pathological conditions due to malnutrition, genetic factors, and certain diseases that affect the aging process. As a result, organ weight and functionality decline with aging [3]. The relationship between organ volume measurements and disease has been the subject of many studies [4]. The pancreas is an organ that has been shown to decrease in size with age. It has a complex histology characterized by a combination of endocrine and exocrine cells [3]. The weight of the pancreas increases until the age range of 30–40, after which the weight of the pancreas gradually decreases. As the weight of the pancreas decreases in the elderly, the likelihood of developing various pancreatic diseases or dysfunctions increases [5]. Atrophy, fatty infiltration, fibrosis, lymphoplasmacytic infiltration, metaplasia, a reduction in endocrine cells, and amyloid deposition are the main changes seen in the pancreas with age [5].

Pancreas volume (PV) is assessed by quantitatively measuring the size of the entire pancreas. CT or magnetic resonance imaging (MRI) is more commonly used for measurement. The normal volume of the pancreas in adults ranges from 71 to 83 cubic centimeters (cm^3^). However, variations in PV are observed in different pathological conditions. For instance, obesity has been demonstrated to result in an increase in PV, while chronic pancreatitis and diabetes mellitus (DM) have been identified as the most prevalent pathological conditions that lead to PV shrinkage [6]. In a large-scale meta-analysis evaluating PV, type 1 DM, and type 2 DM, chronic pancreatitis and ethnic differences were found to be associated with a decrease in PV. It has been suggested that the reduction in PV due to DM is due to the loss of the trophic effect of insulin [6]. In chronic pancreatitis, tissue damage and atrophy caused by recurrent attacks are among the reasons responsible for the decrease in PV [7]. Endocrine disorders of the pancreas most commonly present as DM, while exocrine dysfunction is characterized by malnutrition and vitamin deficiencies with various absorption disorders [8]. CT is the first-line imaging modality for suspected pancreatic disease, and most reported PV values have been determined by CT [9]. The clinical significance of PV measurement is not fully understood. There is a limited number of studies of normal PV measurement in the literature. Generally, studies are performed in pathological conditions or after resection for any reason [10,11,12]. There are studies on diabetes and PV, but these studies include the general population aged 18 years and older [13]. The number of studies evaluating PV only in older patients is limited. Due to variations in age and other individual and social factors, the results obtained from conventional PV measurement studies varied significantly [14]. The determination of PV has important potential in solving clinical problems. Changes in PV may be associated with pathological conditions of pancreatic endocrine or exocrine function [15]. In conclusion, when the existing literature on PV is reviewed, the effects of comorbidities and ethnic differences on the pancreas, the lack of a standardized PV measurement method, and the lack of age-specific classification seem to be the research gaps in this subject.

The aim of this study is to determine the relationship between PV measurement and DM and malnutrition in individuals over 65 years of age, in whom PV is already expected to be low.

## 2. Materials and Methods

**Figure 1:**
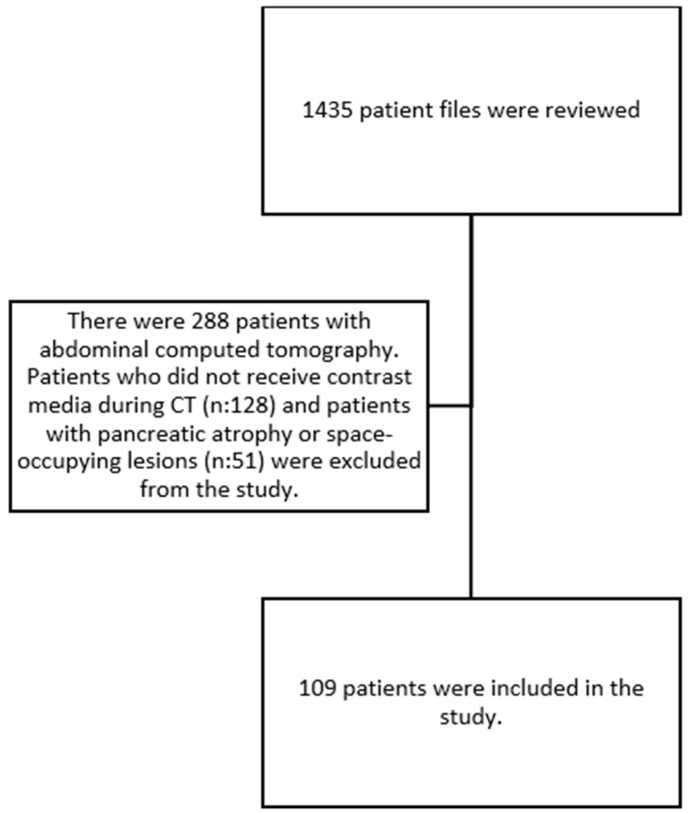
Patient selection flowchart.

This is a retrospective cross-sectional study. Our study was approved by the hospital ethics committee (Date: 11 May 2022, Decision: E2-22-1812). The research was carried out in accordance with the Declaration of Helsinki. Between July 2020 and April 2022, 109 patients over 65 years of age who applied to geriatrics clinics and outpatient clinics were included in the study. The study population comprised patients over the age of 65 with or without diabetes mellitus (DM) who underwent abdominal CT scans for various indications. Patients with a documented history of pancreatic malignancy and chronic pancreatitis, which have been shown to affect PV status, along with patients under the age of 65, were excluded from the study. The patient selection flowchart is shown in Figure 1.

### 2.1. Data Collection

Patient demographics, medical conditions, medications, and laboratory data were retrospectively analyzed from medical records and the hospital information system. To assess pancreatic endocrine function, HbA1c levels, which are a good indicator of glucose regulation and are included in the diagnostic criteria for DM, were evaluated. In all patients, HbA1c levels were measured from blood samples taken in the morning after 8–10 h of fasting. Amylase and lipase levels were determined to evaluate the exocrine functions of the pancreas. Vitamin D, vitamin B12, calcium, total protein, and albumin levels, which are indirect indicators of pancreatic digestion, were measured.

Vitamin B12 levels were measured using the spectrophotometric method, vitamin D levels were measured using the High-Performance Liquid Chromatography (HPLC) method, and biochemical parameters (glucose, ferritin, folate, amylase, lipase, albumin, HDL cholesterol, LDL cholesterol, trygliceride, and ALT) were measured with a modular analyzer using standard laboratory techniques (Cobas 8000 Roche^®^, Mannheim, Germany). Complete blood count was measured with a Sysmex XE2100 (SysmexCorp^®^, Kobe, Japan) automated hematology analyzer.

Nutritional status in elderly patients was evaluated using the Mini Nutritional Assessment Short Form (MNAsf) test. The MNAsf is calculated by evaluating weight loss in the last 3 months, loss of appetite, chewing and swallowing problems, the presence of acute stress or disease history, mobility, neuropsychological problems, and body mass index. It is evaluated over a total of 14 points. A score of 12–14 points indicates normal nutritional status, 8–11 points indicate a person at risk of malnutrition, and individuals who score 0–7 points are considered malnourished [16,17]. PV was evaluated with contrast-enhanced CT.

### 2.2. CT Acquisition

Retrospective analysis was conducted on abdominal CT scans of patients who had their PV measured. CT scans were carried out using GE Revolution CT devices (General Electric, Milwaukee, WI, USA) equipped with 128 and 512 detectors. Contrast material was administered at a rate of 3 mL/s with a dose of 100 mL during the parenchymal phase at 60 s. Scans were performed with the patients in a supine position, covering the distance between the 10th thoracic vertebra and symphysis pubis, with a slice thickness of 0.625 mm at 100 Kv and 110 mAs. Technical term abbreviations were explained upon first use.

### 2.3. Image Analysis

**Figure 2:**
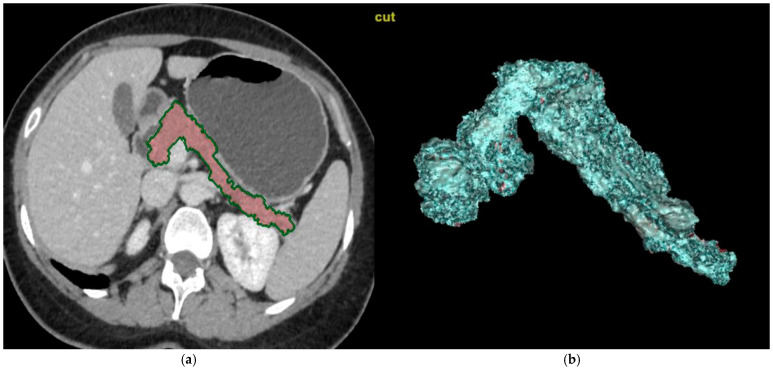
Calculations of the pancreatic volume on CT images. (**a**) The CT scans were transferred to workstations manufactured by GE in the USA, model AW Volume Share 7. Using manually controlled cursors, radiologists outlined the outer boundary of the pancreas during the parenchymal phase on axial images. Fatty tissue and vascular structures, such as the splenic artery and the portal vein, were excluded to the greatest extent possible. (**b**) The total volume of the pancreas was calculated using customized three-dimensional volumetric software (AW4.7 Ext.13 Software, GE, Chicago, IL, USA) on workstations within the designated area.

CT images of the abdomen were reviewed by three radiologists with extensive experience in this field. The CT scans were transferred to workstations manufactured by GE in the USA, model AW Volume Share 7. Using manually controlled cursors, they outlined the outer boundary of the pancreas during the parenchymal phase on axial images. Fatty tissue and vascular structures, such as the splenic artery and the portal vein, were excluded to the greatest extent possible. The total volume of the pancreas was calculated using customized three-dimensional volumetric software (AW4.7 Ext.13 Software, GE, Chicago, IL, USA) on workstations within the designated area (Figure 2a,b).

### 2.4. Statistical Analysis

SPSS version 27 (IBM^®^, Chicago, IL, USA) was used for statistical analysis. Histograms and the Shapiro–Wilk test were used to evaluate whether the variables conformed to normal distribution. Descriptive statistical data were expressed as the mean ± standard deviation and median (minimum–maximum). Parametric/nonparametric tests were used according to their suitability for normal distribution. The Mann–Whitney U test was used to compare demographic and biochemical parameters between the patient and case group. A Chi-square analysis was used to compare nominal data. Spearman’s correlation test was used for a correlation analysis. *p* value less than 0.05 was considered significant.

## 3. Results

The study included 109 participants. Table 1 shows a comparison of the demographic data and MNA scores of all participants and between groups according to the presence of DM based on the Mann–Whitney U test and Chi-square test. The mean age of all participants was M: 77.40 with SD: 7.32. A total of 54.1% of the participants were female. There was no significant difference between those with DM and those without DM in terms of age and gender. The most common comorbidity was HT (64.2%). HT (X^2^(1) = 7.125, *p* = 0.008) and coronary artery disease (CAD) (X^2^(1) = 5.840, *p* = 0.016) were significantly higher in those with DM. There was no significant difference in other comorbidities between the groups. The MNA score for all participants was M: 8.03 with SD: 3.94. The MNAsf score was significantly higher in those with DM (U = 186.500, Z = −2.192, *p* = 0.028).

**Figure 3:**
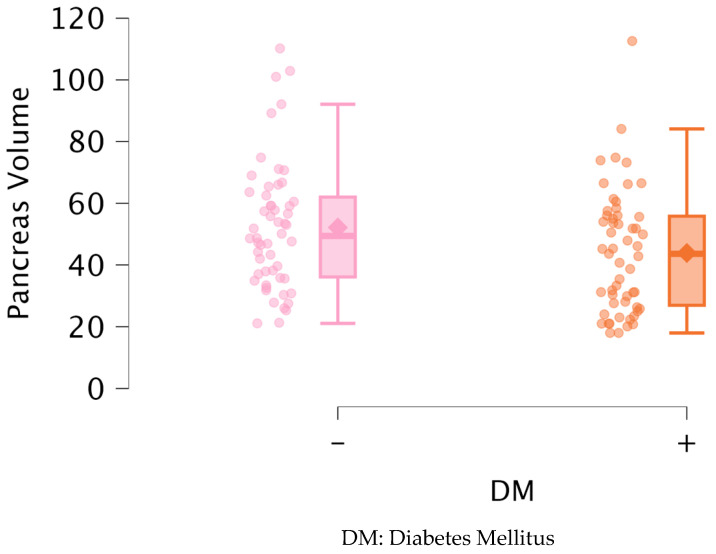
Distribution of pancreas volume between groups.

Table 2 shows a comparison of the biochemical parameters of the groups according to the presence of DM based on the Mann–Whitney U test. Glucose (*p* < 0.001), HbA1C (*p* < 0.001), and triglycerides (*p* < 0.001) were significantly higher and HDL (*p* < 0.001) was significantly lower in patients with DM. PV was also significantly lower in patients with DM (*p* = 0.028) (Figure 3). There was no significant difference between the other parameters.

Table 3 shows a comparison of the PV and biochemical parameters of the groups according to age groups based on the Mann–Whitney U test. Albumin levels were significantly higher in the group aged 65–74 years compared to the group aged 75 years and older (*p* = 0.006). There was no statistically significant difference in the comparison of other parameters.

Table 4 shows a comparison of the PV and biochemical parameters of the groups according to gender based on the Mann–Whitney U test. Vitamin B12 levels were significantly higher in women than in men (*p* = 0.012). There was no statistically significant difference in the comparison of other parameters.

Table 5 shows the correlations between PV, amylase, lipase, albumin, HbA1C, age, and MNAsf score based on Spearman’s correlation analysis. There was a significant positive correlation between PV and MNAsf score (rho (109) = 0.413, *p* = 0.003) and lipase (rho (109) = 0.297, *p* = 0.002). A significant negative correlation was found between age and MNAsf score (rho (109) = −0.337, *p* = 0.016) and albumin (rho (109) = −0.335, *p* < 0.001). Significant positive correlations were found between albumin and MNAsf (rho (109) = 0.428, *p* = 0.002), amylase (rho (109) = 0.194, *p* = 0.043), and HbA1C (rho (109) = 0.249, *p* = 0.010). Amylase was significantly positively correlated with lipase (rho (109) = 0.520, *p* < 0.001) and significantly negatively correlated with HbA1C (rho (109) = −0.289, *p* = 0.003). There was no significant correlation between the other comparisons.

## 4. Discussion

PV varies across the lifespan. Studies have shown that PV generally decreases after the age of 60 and that there is a negative correlation between age and PV [4,15,18]. These studies usually include the general population aged 18 years and older. There are no studies in the literature evaluating DM and PV only in patients aged 65 years and older [19,20]. In our study, we evaluated the population over 65 years of age in terms of pancreatic volumes according to the presence of DM. As expected, PV was lower compared to the general population. When we divided the patients according to the presence of DM, PV was lower in the diabetic group. No significant correlation was found between PV and HbA1c levels, which are important in DM follow-up. In a study by Oz et al. comparing 53 type 2 DM patients with a healthy control group, PV was found to be significantly lower in the DM group. While an association was found between PV and HOMA-IR, no evaluation was made in terms of Hba1c [19]. Noda et al. compared DM, prediabetes, and healthy patients in their PV studies with MRI and found a relationship between HbA1c and PV [21]. Iwamoto et al. studied PV in 58 patients with type 2 DM and 7 patients without DM using MR imaging and suggested that an increased pancreatic fat content and a serrated pancreatic limbus were responsible for the decrease in PV [22].

The relationship between aging and DM has been demonstrated in many studies [23,24,25]. DM risk increases with age, but the mechanism remains unclear [26]. The International Diabetes Federation (IDF) estimates that the number of people with DM aged 65–99 years was 136 million people in 2019 [27]. There are also studies showing that HbA1c levels increase with aging independent of DM [28]. In our study, no significant difference was found between the groups with and without DM in terms of age and HbA1c. We thought that this might be due to the fact that the sample group consisted of individuals aged 65 years and older. A study of 240 Chinese patients aged 18–79 years showed that PV increased until the age of 50 years and then began to decrease. PV was found to be 73.00 ± 18.48 cc in individuals between 60 and 69 years of age and 67.10 ± 21.59 cc in those between 70 and 79 years of age [9]. These values are higher than those in our study. When we differentiated according to age in our study, the PV was 51.77 ± 21.66 (cm^3^) in the 65–74 age group and 45.66 ± 19.60 (cm^3^) in the 75 years and older age group. There was no difference between these two age groups in terms of PV. This suggests that there may be different results depending on measurement techniques, ethnic differences, and sample size. Among the studies evaluating changes in PV and its structure with age, a 1983 study involving 915 patients and ultrasonographic evaluation found a positive correlation between age and PV [29]. Migdalis et al. found a decrease in PV with age in a CT-based study comparing 84 non-insulin-dependent DM patients and 80 controls [30], while Geraghty et al. found no change in PV with age in a study of 149 patients [4]. These studies are quite heterogeneous in terms of different measurement methods, racial differences, and the size of the sample groups, making it difficult to make generalizations about the subject [31].

A multitude of conditions have been identified as affecting PV. One such condition is non-pancreatic malignancies. In particular, chemotherapeutic agents have been shown to have effects on pancreatic function and volume. Phillip et al. measured PV in malignant patients treated with sorafenib and bevacizumab and found a significant decrease in PV [32].

In our study, there was a total of nine patients with non-pancreatic malignancy. The treatments they received for malignancy were unknown. Therefore, a comparison with PV could not be made. In cancer patients who already have nutritional problems, revealing pancreatic dysfunction may also be instructive in terms of non-cancer treatment plans and chemotherapeutic selection. The necessity for more comprehensive studies on this subject is evident.

The pancreas is an important organ involved in the digestion of proteins, carbohydrates, and fats. The main pancreatic enzymes responsible for digestion are amylase, lipase, and proteases. Exocrine dysfunction of the pancreas can lead to fat-soluble vitamin deficiency, steatorrhea, osteoporosis, and malnutrition [3,33,34]. Among laboratory parameters, abnormalities may be observed in vitamin levels, calcium, and albumin levels [8,35]. Recent studies have demonstrated that, among fat-soluble vitamins, particularly vitamin D and vitamin E, when present in sufficient amounts or when administered as supplements in cases of deficiency, have been shown to exert a beneficial effect on bone health and cognitive functions in elderly individuals [3]. Since pancreatic exocrine insufficiency in the elderly is not well studied, most of the data on the consequences of pancreatic exocrine insufficiency come from studies on chronic pancreatitis and cystic fibrosis [34]. Age-related changes, fibrosis, and atrophy of the pancreas can result in enlargement of the pancreatic duct and chronic pancreatitis. Chronic pancreatitis is an important cause of exocrine dysfunction. Another change observed with age is a decrease in pancreatic perfusion. There are studies suggesting that this decrease in perfusion due to atherosclerotic changes is associated with mild chronic pancreatitis [36]. Chronic pancreatitis is especially important in elderly patients as it can cause digestive problems and malnutrition [36]. Malnutrition can be an important cause of mortality and morbidity, especially in elderly patients. The MNAsf test is a simple and practical test used to screen for malnutrition in elderly patients [37,38]. In our study, a significant positive correlation was found between PV and MNAsf scores and lipase levels. Higher PV seems to be characterized by better nutritional status. Adequate exocrine function will protect the patient from many complications, including vitamin deficiencies and osteoporosis [8]. This suggests that the contribution of the pancreatic volume to malnutrition in the elderly, especially through exocrine functions, should be evaluated. The extant literature on this subject is limited [3]. Pancreatic enzyme deficiency may be an important cause of malnutrition. In the presence of malnutrition that does not improve despite treatment in elderly patients, evaluation for pancreatic enzyme replacement may be considered [39].

In conclusion, PV was found to be low in diabetic elderly individuals evaluated with CT in our study. Existing studies include patients aged 18 years and older, and studies with detailed evaluations over the age of 65 years seem to be insufficient. Furthermore, an examination of the extant literature revealed significant heterogeneity among studies conducted with PV. The PV measurement method and the parameters compared are among the primary factors contributing to this heterogeneity [6,36,40,41]. It is thought that more large-scale prospective studies on the subject are needed.

Our study had several limitations. First, it was retrospective, the sample size was small, and it was a single-center study. Another limitation was the inability to establish a cause-and-effect relationship due to the cross-sectional study design. In light of the extant research demonstrating the impact of obesity and body mass index (BMI) on PV, the absence of these parameters in the present study constitutes a significant limitation [40]. While PV was measured, parameters that may affect clinical outcomes, such as the pancreatic fat content and degree of fibrosis, could not be assessed. Due to the retrospective study design, objective diagnostic tests for malnutrition and more specific laboratory tests, such as prealbumin and fecal elastase, could not be evaluated [42]. Considering other PV studies evaluating different ethnic groups, the fact that this study only evaluated the Turkish community was another limitation [43]. Although the PV measurement method was selected as the most commonly used CT in the literature, the accuracy and precision of the method should be examined.

## 5. Conclusions

Studies have revealed that the most significant factor affecting pancreatic volume is age. In our study, we obtained similar results to those in the literature. With physiological aging, the pancreas is expected to decrease in size as in every organ. DM is also an important factor contributing to this shrinkage. Physiological and pathological conditions caused by the volume changes that occur with aging have not yet been fully elucidated. Our study highlights the importance of determining the clinical effects of pancreatic volume in the geriatric population, where organ atrophy is expected. A review of the extant literature on PV revealed several research gaps, including the effects of comorbidities and ethnic differences on the pancreas, the lack of a standardized PV measurement method, and the lack of age-specific classification. Therefore, we believe that more comprehensive studies are needed to clarify the clinical implications of pancreatic volume on our diagnostic and therapeutic decisions.

## Figures and Tables

**Table 1:** Comparison of demographic and clinical data of all participants and according to presence of diabetes mellitus based on Mann–Whitney U test and Chi-square test (N = 109).

	All Patients (N = 109)	DM (−) (n = 54)	DM (+) (n = 55)	*p* Value
Age (year) *	77.40 ± 7.32	78 ± 7.76	76.81 ± 6.89	0.470
Gender, F **	59 (54.1)	25 (46.3)	34 (61.8)	0.104
Comorbidities (+) **				
HT	70 (64.2)	28 (51.9)	42 (76.4)	0.008
CAD	34 (31.2)	11 (20.4)	23 (41.8)	0.016
Dementia	30 (27.5)	12 (22.2)	18 (32.7)	0.220
COPD	17 (15.6)	11 (20.4)	6 (10.9)	0.173
CVD	14 (12.8)	8 (14.8)	6 (10.9)	0.542
CHF	13 (11.9)	5 (9.3)	8 (14.5)	0.395
Non-Pancreatic Malignancy	9 (10.5)	7 (14)	2 (5.6)	0.207
MNAsf score *	8.03 ± 3.94	7.15 ± 3.70	9.66 ± 3.94	0.028

* Mean ± sd, ** N (%). HT: hypertension, CAD: coronary artery disease, COPD: chronic obstructive pulmonary disease, CVD: cerebrovascular disease, CHF: congestive heart failure, MNAsf: Mini Nutritional Assessment Short Form.

**Table 2:** A comparison of the biochemical parameters of the groups according to the presence of diabetes mellitus based on the Mann–Whitney U test (N = 109).

	All Patients (N = 109)	DM (−) (n = 54)	DM (+) (n = 55)	*p* Value
Glucose (mg/dL)	125.86 ± 52.75	104.56 ± 25.50	146.38 ± 63.35	<0.001
HbA1c (%)	6.57 ± 1.80	5.58 ± 0.63	7.50 ± 2.04	<0.001
Vitamin D (nmol/L)	47.80 ± 33.31	45.90 ± 32.68	49.62 ± 34.12	0.581
Vitamin B12 (ng/L)	552 ± 395	543 ± 432	562 ± 358	0.311
Ferritin (µg/L)	180 ± 358	223 ± 435	136 ± 252	0.095
Folat (ng/L)	11.96 ± 9.79	12.90 ± 11.51	11.06 ± 7.78	0.937
Amylase (U/L)	70.14 ± 45.40	72.92 ± 45.04	67.41 ± 46	0.202
Lipase (U/L)	37.10 ± 18.94	38.60 ± 19.94	35.65 ± 17.98	0.363
Hemoglobin	11.46 ± 2.14	11.41 ± 2.16	11.51 ± 2.13	0.961
Albumin (g/dL)	3.68 ± 0.73	3.64 ± 0.71	3.73 ± 0.74	0.418
HDL cholesterol	40.89 ± 17.04	46.77 ± 19.12	35.12 ± 12.38	<0.001
LDL cholesterol	95.33 ± 34.89	98.33 ± 37.03	92.33 ± 32.68	0.258
Triglyceride	131.80 ± 50.07	115.88 ± 45.89	147.42 ± 49.46	<0.001
ALT	20.10 ± 12.64	19.90 ± 13.97	20.29 ± 11.31	0.466
PV (cm^3^)	47.96 ± 20.52	52.12 ± 20.62	43.87 ± 19.76	0.028

Mean ± sd. DM (+): patients with diabetes mellitus, DM (−): patients without diabetes mellitus, HbA1C: glycolyzed hemoglobin A1c, HDL: high-density lipoprotein, LDL: low-density lipoprotein, ALT: alanine aminotransferase, PV: pancreas volume.

**Table 3:** A comparison of the PV and biochemical parameters of the groups according to age groups based on the Mann–Whitney U test (N = 109).

	65–74 Years	≥75 Years	*p* Value
Diabetes Mellitus (+) *	22 (40)	33 (60)	0.604
HbA1c (%)	6.88 ± 2.05	6.38 ± 1.62	0.245
Glucose (mg/dL)	128.29 ± 57.44	124.37 ± 50.05	0.562
Vitamin D (nmol/L)	47.91 ± 31.30	47.73 ± 34.71	0.751
Vitamin B12 (ng/L)	458 ± 267	608 ± 446	0.079
Hemoglobin	11.96 ± 2.01	11.16 ± 2.17	0.061
Albumin (g/dL)	3.93 ± 0.64	3.53 ± 0.74	0.006
Total protein	6.31 ± 0.70	5.96 ± 0.88	0.050
PV (cm^3^)	51.77 ± 21.66	45.66 ± 19.60	0.201

Mean ± sd, * n (%). HbA1C: glycolyzed hemoglobin A1c; PV: pancreas volume.

**Table 4:** Comparison of PV and biochemical parameters of groups according to gender based on Mann–Whitney U test (N = 109).

	Male	Female	*p* Value
Diabetes Mellitus *	34 (61.8)	21 (38.2)	0.104
HbA1c (%)	6.29 ± 1.45	6.79 ± 2.02	0.426
Glucose (mg/dL)	123.78 ± 42.97	127.65 ± 60.24	0.637
Vitamin D (nmol/L)	42 ± 31.92	52.37 ± 33.94	0.071
Vitamin B12 (ng/L)	446 ± 272	641 ± 457	0.012
Hemoglobin	11.61 ± 2.29	11.34 ± 2.01	0.397
Albumin (g/dL)	3.71 ± 0.72	3.66 ± 0.74	0.677
Total protein (g/dL)	6.05 ± 0.85	6.12 ± 0.82	0.556
PV (cm^3^)	51.16 ± 21.35	45.25 ± 19.56	0.170

Mean ± sd, * n (%). HbA1C: glycolyzed hemoglobin A1c; PV: pancreas volume.

**Table 5:** Correlations between PV, pancreatic enzymes, age, albumin, HbA1C, and MNA score based on Spearman’s correlation test (N = 109).

	PV	Age	MNAsf	Albumin	Amylase	Lipase	HbA1c
Age	−0.145	-					
MNAsf	0.413 *	−0.337 *	-				
Albumin	0.180	−0.335 **	0.428 *	-			
Amylase	0.170	−0.055	0.058	0.194 *	*-*		
Lipase	0.297 *	−0.042	−0.050	0.129	0.520 **	*-*	
HbA1c	−0.072	−0.140	0.250	0.249 *	−0.289 *	−0.187	-
Vitamin D	−0.082	0.007	0.133	0.086	0.147	0.142	−0.081
Vitamin B12	−0.078	0.155	−0.040	−0.197 *	−0.093	0.003	0.001

* *p* < 0.05; ** *p* ≤ 001. MNAsf: Mini Nutritional Assessment short form, HbA1C: glycolyzed hemoglobin A1c, PV: pancreas volume.

## Data Availability

The data supporting the findings of this study can be obtained, but there are restrictions on the availability of these data; these data were used under license for the current study and are therefore not publicly available. However, the data can be obtained from the corresponding author upon reasonable request.

## Abbreviations

PV	Pancreas volume
HbA1c	Glycolyzed hemoglobin A1c
DM	Diabetes mellitus
CT	Computed tomography
HDL	High density lipoprotein
MNAsf	Mini Nutritional Assessment Short Form
CAD	Coronary artery disease
HT	Hypertension
MRI	Magnetic resonance imaging
BMI	Body mass index
